# The Intertinal Diseases of Infancy and Childhood

**Published:** 1890-11

**Authors:** 


### Book Reviews.

THE INTERTINAL DISEASES OF INFANCY AND CHILDHOOD. PHYSIOLOGY, HYGIENE, PATHOLOGY AND THEROPEUTICS. By A. Jacobi, M. D. Published by Geo. S. Davis. (Physicians' Leisure Library). Cloth 50 cents, Paper 25 cents.

This work of Dr. Jocobi is decidedly a very comprehensive treatise upon this very interesting and important branch of the practice of medicine, viz: The digestive disorders of infants. It consists of two volumes, Nos. V. and VI., of Geo. S. Davis' Physicians' Leisure Library Series. In vol. I. (No. V.) the author gives us his views very clearly upon all articles that may be used as food for infants. The manner of feeding, both during infancy and period succeeding infancy.

Dr. Jacobi begins the first chapter by saying: "In many of the digestive disorders of infants, the best preventive, and often curative aid is the breast milk of mother or wet nurse." As a substitute for breast-milk, he prefers boiled cow's milk, and permits condensed milk, when good milk (cow's) cannot be obtained. He advocates a plentiful addition of water to the milk, and adds that this nutriment (milk and water) should be mixed with cane-sugar, and table-salt, he says that salt is "of great physiological importance in the interest of tissue change in general." He says that some starch is digested at the very earliest age, besides being nutritious, it serves to dilute cow's milk, reduce its percentage of casein, and renders it digestible. He prefers barley and oatmeal to supply the amount of starch necessary—recommends barley meal for children who have a tendency to diarrhoea, and oatmeal to those who have a tendency to constipation—and mentions also the utility of gum arabic, and gelatin as an addition to cow's milk—and to children's diet—"not only do they fulfill the indications of diminishing and distributing the particles in cow's milk, but they also officiate as a means of direct nourishment by preventing waste."

Dr. Jacobis says infants should be fed from a bottle, and that his experience leads him to conclude that the new-born infant should be fed from eight to ten times daily—after it is four months old, five daily nursings will suffice. He adds: "If a child which is healthy and not spoiled awakens at night it needs and desires nothing but a drink of water, or of thin barley water, without milk."

On page 88 of chapter, on Mouth, we find the following: "It must be remembered that within the first year the mortality among infants is greatest from diseases of the organs of respiration, undoubtedly in consequence of the fact that during that period, infants are more exposed to atmospheric changes than in earlier life—this is one of the reasons why, at the time of dentition, not in consequence of dentition, pulmonary diseases are frequent." From this we learn that dentition is not the cause of disease, as has often been claimed, but merely the period at which infants are more susceptible to conditions which induce diseases of the digestive organ—and to atmospheric changes which induce pulmonary diseases. The diarrhoea occurring during dentition cannot always be attributed to teething.

Dr. Jacobi remarks, "that the large majority of infants who are either at the breast, or whose artificial food is well selected, do not suffer from diarrhoea while going through the process of dentition." He concludes that the number of diseases which have been attributed to dentition is due to the limited diagnostic power of the practitioner.

The last chapter of vol. 1, No. v. is devoted to the Stomach, Digestion, and the diseased condition affecting the stomach.

Vol. II, openes with a chapter on Intestinal Digestion—in which is given the anatomy, and functions of the infantile intestines and the other abdominal organs connected with the digestive system. Speaking of the pancreas, Dr. Jacobi says: "The pancreas performs its functions only while the intestinal fluids are alkaline, and this alkaline reaction depends upon the presence of phosphate of sodium. When there is acid in the intestines, the pancreas is prevented from performing its function, and the formation of bone is delayed." This condition of things gives rise to the disease known as Rhachitis, and thus we find Rhachitis to follow chronic digestive disorders.

In acute intestinal catarrh, Dr. Jacobi prefers, as an antifermentative, resorcin, iv–x grains a day in solution, or as a constituent of powders with bismuth chalk, or opium. Of diet, in this condition, (acute intestinal catarrh), he says: "No raw milk, no boiled milk, no milk at all, in bad cases—in vomiting, and severe diarrhoea, total abstinence for from one to six hours, afterwards, teaspoon doses of a mucilaginous or Farinaceous decoction."

Short chapters are devoted to Tuberculosis, Ulceration of the Stomach, Perityphlitis, Paratyphlitis, Proktitis, Prolapsus of the Rectum, Intersusception, Malformation of the Intestines, Dysentery and Parasites."

A chapter on Fissure of the Anus, closes this volume. Dr. Jacobi, quoting Kjellberg, says: "9098 children were presented in the Dispensary of Stockholmn; in 128 there was fissure of the anus; 60 were boys, 68 girls; the majority less than a year old; 23 less than four months. Diagnosis—pulling hard upon the anus to the right or left, it becomes perceptible, as a narrow cut, red and clear, or sometimes grayish, and ulcerated one-half, two, or even five lines in length. Very seldom does it extend beyond the sphinter, the slightest touch is exceedingly painful, and the pain at defæcation is very intense. The "proper treatment consists in dilatation of the sphincters—the best and speediest method is forcible instantaneous dilation without anaesthetia."

These volumes are replete with that sound judgement and knowledge which comes from a ripe experience—they are written in a terse style and all the deductions are conclusive, and evince a thorough acquaintance with the subject.

Dr. Jacobi ranks high as an authority on the diseases of children. He has written valuable articles relative to "Infant Diet," and on "Hygene and Public Health;" and we feel confident that these volumes will meet with the hearty endorsement of the entire profession. E. V. J.

---

AN EARLY SYMPTOM OF LOCOMOTOR ATAXIA.—Dr. Heinrich Weiss describes (*Wien. Med. Press*) the case of a book-keeper whose first symptom was an uncertainty in stepping backward, in whom characteristic manifestations of locomotor ataxia subsequently developed. The importance of this initial symptom was pointed out by Althaus in 1884, in reporting the case of a painter who noticed it in himself as he backed away from his easel.—*Deutsche Medizinal Zeitung.*

